# Evaluation of phytochemicals and essential oils of *Cupressus semprevirens* in controlling cattle tick *Rhipicephalus annulatus* (Acari: Ixodidae)

**DOI:** 10.1186/s12870-025-06222-5

**Published:** 2025-03-11

**Authors:** Mai Ahmed Taha, Asmaa Ali Baioumy Ali

**Affiliations:** 1 Botany Department, Faculty of Science, Ain Shams University, Abbassia, Cairo, Egypt; 2 Zoology Department, Faculty of Science, Ain Shams University, Abbassia, Cairo, 11566 Egypt

**Keywords:** Acaricidal activity, *Cupressus sempervirens*, Essential oils, Fatty acids, Phytochemicals, *Rhipicephalus annulatus*

## Abstract

**Background:**

*Cupressus sempervirens* is one of the conifer plants, that is used as an antimicrobial, antioxidant, anthelminthic, and many other health purposes. *Rhipicephalus annulatus* is one of the hard tick genera affecting the production and health of domestic animals in Egypt. Extensive use of chemical acaricides in the management of ticks caused acaricide resistance, environmental contamination, residues in meat and milk, and harmful effects on non-target species. For these reasons, there is an urgent need to create efficient, environmentally friendly acaricides. This work aimed to assay the essential oils and establish the phytochemical analysis of *C. sempervirens* extract, its effects against the semi-engorged females of *R. annulatus*, and discuss their possible control effects.

**Results:**

Using the spray-dip method by *C. sempervirens* ethanol, methanol, distal water, and chloroform extracts at different concentrations (10–50%), revealed a decrease in the percentage of mobile female *R. annulatius* ticks, and increased mortality proportionally with days after treatments (14 days) and/or extract concentrations (10–50%). The ethanol extracts showed their strongest acaricidal effect, where the female mortality percentage reached 100% using all concentrations at the end of the examined period. In addition, the estimated LC_50_ and LC_95_ of *C. sempervirens* ethanolic extract were recorded as the lowest values (12.2% and 17%, respectively) after 14 days of treatment compared with other extract types. The 50% ethanolic extract of *C. sempervirens* (the most effective one) revealed the presence of bioactive metabolites i.e. flavonoids, tannins, and carbohydrates (TSS). Also, its total antioxidant capacity and potential free-radical activity (DPPH) were estimated. Using GC-MS, the extracted oil revealed the presence of four major compounds i.e., Eicosapentaenoic acid (50.85%), 10,12-Docosadiynedioic acid (27.58%),10-Undecynoic acid (14.28%) and Palmitic acid (5.42%). The efficiency of all phytochemicals and essential oils was discussed in the current study.

**Conclusion:**

The phytochemicals and essential oils found in *C. sempervirens* could enhance our understanding and help in developing potential strategies for controlling ticks in general and for *R. annulatus*, in particular, using environmentally friendly agents.

## Background

Gymnosperms are the first seedling plants consisting of 1079 species in 12 families and 4 phyla [1, 2]. Coniferophyta (Conifers) is the dominant phylum of gymnosperms. *Cupressus sempervirens* L. var. *stricta* (syn. var. *horizontalis*) is one of the conifer evergreen plants, up to 35 m tall with loose branches, dense, dark green leaves (2–5 mm long), and a conical crown [3, 4]. It is known as the Mediterranean cypress and belongs to the Cupressaceae family [5]. It is an aromatic, medical, ornamental widely cultivated plant, mainly found throughout the whole Mediterranean region [5]. Being a pioneer plant, it overgrows on most soil types while it is young, even compacted and rocky ones [6, 7].

*Cupressus sempervirens* is used traditionally in folk remedies as antiseptic, antipyretic, anthelminthic, antidiarrhoeic, antirheumatic, astringent, and vasoconstrictive purposes [5, 8]. It is also reported to possess many biological activities for human health as antimicrobial activity, antiprotozoal activity, insecticidal activity (repellent or lethal activity), anticancer, antioxidant effect, anticoagulant activity, hepatoprotective activity, wound healing, anti-inflammatory activity, and neurobiological activity [5, 8]. Various classes of phytochemical compounds have been reported in its different parts, including flavonoids, terpenes, catechins, proanthocyanidins, essential oils, phenolic acids, and fatty acids [5, 8–10].

Ticks are the most common ectoparasites that infest animals and humans through hematophagous nutrition, and pathogens and toxins transmission [11, 12]. They can cause babesiosis, theileriosis, rickettsial diseases, dermatophilosis in livestock, Lyme disease, Kyasanur forest disease, and tick-borne encephalitis in humans [13, 14].

*Rhipicephalus* is one of the most economically important hard tick genera affecting domestic animals [15, 16]. *Rhipicephalus annulatus* (Say 1821) is a one-host tick distributed in tropical and subtropical regions [12]. In Egypt, it is the most common cattle-infesting tick [17]. It also can affect sheep, horses, donkeys, dromedary camels, buffalo, and sheep [18, 19]. Its infestation harms animal production and health through sucking blood, decreasing body weight and milk productivity, stress induction, and immune dysfunction, in addition to transmission of hemoparasites such as *Babesia* spp., and *Anaplasma* spp [20–22].

Chemical acaricides are widely used in the management of ticks because they are readily available and simple to apply [17, 23]. In addition to acaricide resistance, environmental contamination, and residues in meat and milk, this management technique has harmful effects on nontarget species [24, 25]. Moreover, *R. annulatus* can quickly acquire resistance traits to a variety of acaricide classes [23]. For these reasons, there is an urgent need to create efficient, environmentally friendly acaricides.

Now, plants are considered suitable alternatives for tick control as they are safe for public health and the environment [26]. Since no research has been carried out on the efficiency of essential oils (fatty acids and their derivatives: FAs) and other phytochemical components obtained from *C. sempervirens* L. var. *stricta* against *R. annulatus* in Egypt, the goal of this study was to establish the phytochemical analysis of *C. sempervirens* extract and its effects against the semi-engorged females of *R. annulatus*. Additionally, the aim was to determine the plant’s essential oils and understand how these phytochemicals connect to the management of *R. annulatus*.

## Methods

### Plant collection and preparation

Fresh, healthy leaves were collected from mature trees located in the gardens of Ain Shams University campus, Cairo, Egypt (GPS coordinates 31°17′7′′ E and 30°4′35′′ N), 29 m above sea level, which were cultivated as ornamental trees and growing on wet, clay lands. Then they transferred to Botany Laboratory, Botany Department, Faculty of Science, Ain Shams University. According to Vidakovic [3], Farjon [4], and Nehdi [27], the plant materials have been identified as *Cupressus sempervirens* L. var. *stricta* (Pinophyta, Cupressales, Cupressaceae). The leaves were thoroughly washed many times with distilled water to remove any surface dirt or impurities. The clean leaves were air-dried at room temperature in shade for 14 days then oven dried at 60 °C for 3 days to remove excess moisture. After drying, the leaves were ground into a fine powder using an electric blender (Arion, 250 W). The powdered plant was kept at -4 ˚C until the extraction preparation.

### Preparation of polar and nonpolar plant extracts

The extraction method was modified from Selim et al. [28] and Altemimi et al. [29]. It was performed in Central Laboratory, Faculty of Science, Ain Shams University, Cairo, Egypt. Plant powder was portioned and suspended for 48 h in different solvents of ethanol, methanol, and distal water (as polar solvents), and chloroform (as a nonpolar solvent). Each extract was adjusted at concentrations of 10–50%, with a total of 20 extracts. They were centrifuged at 400 rpm for 10 min and the supernatants were evaporated in open air at room temperature for 10 days. The dry residue was dissolved in distilled water to obtain different concentrations of the crude extract.

### Quantitativeanalysisofphytochemicals

The quantitative analysis of the phytochemicals was performed using 50% ethanolic extract of the plant material to estimate tannins and flavonoids (as secondary metabolites), carbohydrates, antioxidant activity (through DPPH radical), and total antioxidant capacity. All methods were implemented in Central Laboratory, Faculty of Science, Ain Shams University, Cairo, Egypt. The mean ± standard error (SE) was employed to express the estimated parameters (done in triplicate) using Excel Microsoft 365 (2010).

#### Carbohydrate analysis

The assay for soluble sugar was conducted using the procedure described by Blakeney and Mutton [30]. Anthrone reagent (10 mL) and 2 mL of dissolved residue were combined, heated to a boil water bath for 20 min, cooled, and the absorbance at 620 nm was measured with a UV-Vis spectrophotometer. Using the standard curve of glucose, the carbohydrate content was determined and represented as mg/ g of the dry weight (DW).

#### Extraction and estimation of flavonoids

The total flavonoid content of the plant extract was determined using the aluminum chloride colorimetric method [31, 32] with some modifications. Following the plant sample’s flavonoid extraction, 1 mL of the extract was mixed with 1.5 mL of methanol, 0.1 mL of a 10% aluminum chloride solution, 2.8 mL of distilled water, and 0.1 mL of 1 M potassium acetate. A UV-Vis spectrophotometer was used to detect the absorbance at 415 nm following a 30-minute incubation period at room temperature. The total flavonoid content was calculated using the standard curve equation and expressed as mg quercetin equivalents (QE)/g of the DW.

#### DPPH (1, 1-diphenyl-2-picrylhydrazyl) radical scavenging assay

According to Yamaguchi et al. [33] with slight modifications, the DPPH radical scavenging activity of the extract was determined. The 1.5 mL of ethanolic extract was mixed with 0.5 mL methanolic solution of the DPPH (0.1mM) and incubated in the dark at room temperature for 30 min. The absorbance was measured at 517 nm using a UV-Vis spectrophotometer. The percentage of DPPH scavenging activity was calculated as follows;

$$\begin{array}{l}\\{\bf{DPPH}}{\rm{ }}{\bf{radical}}{\rm{ }}{\bf{scavenging}}{\rm{ }}{\bf{activity}}{\rm{ }}\left( \% \right) = \\\left[ {\left( {{\bf{A0}}-{\bf{A1}}} \right)/{\bf{A0}}} \right]{\rm{ }} \times {\bf{100}}\end{array}$$

Where A0 is the absorbance of the control (the DPPH blank solution without extract) and A1 is the absorbance of the sample.

#### Estimation of total antioxidant capacity (TAC)

The phosphomolybdenum method [34] was applied to estimate the total antioxidant capacity of the plant extract with slight modifications. Ascorbic acid was used as the standard to create a standard curve. A UV-Vis spectrophotometer was employed to detect the absorbance at 695 nm. Using the standard curve equation, the total antioxidant capacity was determined and represented as mg of ascorbic acid equivalents (AAE)/g of the plant material’s DW.

#### Measurement of tannins

The tannins of the plant extract were determined using the Folin-Ciocalteu method [35]. The total tannins were calculated using the vanillin reagent as reported by Price et al. [36]. A spectrophotometer (Shimadzu UV265, Japan) was used to detect the absorbance at 500 nm. The tannin content was calculated using the standard curve equation and expressed as mg tannic acid equivalents (TAE)/g of the DW.

### Oil extraction and recovery

Plant oils were extracted using the Soxlet apparatus according to López-Bascón and Luque de Castro [37], with slight modifications in Central Laboratory, Faculty of Science, Ain Shams University, Cairo, Egypt. The extraction thimble was filled with 5 g of plant powder inside a filter paper, and the round-bottom flask was filled with petroleum ether as an organic solvent, covering the extraction thimble. The solvent in the round-bottom flask was heated to its boiling point (60 °C), causing it to evaporate and rise through the condenser. The extraction process was initiated and continued for 8 h, to ensure maximum extraction efficiency. Extracting oil components (FAs) from the plant material separated from the solvent by evaporation using a rotary evaporator under reduced pressure as an oil recovery method.

### Oil analysis

The separation, detection, and identification of *C. sempervirens* oil components were done in the Central Laboratory, Faculty of Science, Ain Shams University, Cairo, Egypt. They were subjected to Gas Chromatography-Mass Spectrometry (GC-MS) analysis (Agilent Technologies 7890B GC Systems with 5977 A Mass Selective Detector) [38].

#### Chromatographic separation and Mass Spectrometric (MS) detection

A capillary column (HP-5MS Capillary; 30.0 m X 0.25 mm ID X 0.25 μm film) was employed, and helium was injected with 1 µl at a pressure of 8.2 psi. The sample was examined with the column kept at 50° C for 3 min following injection. Split mode injection was carried out at 300° C with a split ratio of 1:1. The MS scan’s m/z range was 50–550 atomic mass units (AMU), with an electron impact (EI) ionization of 70 eV and a solvent delay of 8.0 min.

#### Silylation process

N, O-Bis(trimethylsilyl)trifluoroacetamide (BSTFA) containing trimethylchlorosilane is the silylation agent. After extraction, 250 uL of BSTFA + quantity is added to the sample, the reaction is carried out by injecting the sample into a GC/MS under the specified conditions [39, 40].

#### Data analysis

The chemical constituents were analyzed using appropriate mass fragmentations and The NIST mass spectral search software for the NIST/EPA/NIH mass spectral library Version 2.2 (Jun 2014).

### Tick collection and identification

In Kerdasa center, Kerdasa, Imbaba, Giza Governorate, Egypt, adult *Rhipicephalus annulatus* ticks were collected from naturally infested cattle (*Bos taurus*) (GPS coordinates 30°01’51.9” N 31°06’41.3” E). They were classified as semi-engorged females and fed males in the Invertebrate Laboratory, Zoology Department, Faculty of Science, Ain Shams University, Cairo, Egypt, based on their identification following [41]. Specimens were stored in glass vials with rubber band-secured gauze covers, within 24 h of collection, at 28 ± 1 °C and 75 ± 5% relative humidity until the experiment time.

### Tick treatment

Using the spray-dip method [42], semi-engorged females were treated with 10–50% of each plant extract (ethanol, methanol, water, chloroform) in a 9 cm Petri dish, 25 cm far from the surface, at Invertebrate Laboratory, Zoology Department, Faculty of Science, Ain Shams University, Cairo, Egypt. Three trials were done for untreated (control) and treated groups, each with 10 ticks. All specimens were reared in glass vials and kept inside the incubator (28 ± 1 °C and 75 ± 5% RH). Treated ticks were compared with untreated ones and observed for 14 days. The current investigation included 630 semi-engorged female tick specimens, of which 30 were left untreated and 600 were treated with various plant extracts at different concentrations.

### Tick biological parameters

#### Mobility and mortality

Tick specimens were examined daily for mobility and mortality. The percentage of mobile ticks determined mobility during the examined period, while mortality was for dead ones [43]. Ticks were considered dead if they didn’t move their legs.

#### Lethal concentrations

Fourteen days after tick treatment, lethal concentrations (LC) of all used plant extracts were determined as LC_50_ (lethal concentration at which the mortality rate reached 50%) and LC_95_ (lethal concentration at which the mortality rate reached 95%).

#### Parameters analysis

IBM SPSS (Statistical Package for the Social Sciences) Software-Java compatible (Version 25) was used to analyze the relationships between treated groups in contrast with untreated ones. The data values were expressed as the mean, and standard deviation, and error. For comparisons, data was evaluated using one-way ANOVA followed by Tukey’s test [44] for multiple comparisons. When significant differences (*p* < 0.05) were considered, the effect of several plant extracts on ticks was assessed. Lethal concentrations (LC) were estimated by applying Probit analysis using Probitvb6 [45].

#### Efficacy

According to Wang et al. [46], the efficacy of each plant extract (E) on ticks after 14 days of treatment is as follows;

$$\:\text{E}\:=\:[\text{B}-\text{T}/\:\text{B}]\:100$$

where B is the mean number of surviving ticks in the control group and T in the treated one.

All experimental procedures were carried out in compliance with the standards that the Faculty of Science, Ain Shams University, Cairo, Egypt’s Research Ethics Committee had approved (Code: ASU-SCI/ZOOL/2024/10/6).

## Results

### Quantitative analysis of phytochemicals

Notably, the ethanolic extract of the studied plant at a concentration of 50% had the highest acaricide effect (as shown in the subsequent results), so a quantitative phytochemical analysis was performed on this extract.

**Fig. 1:**
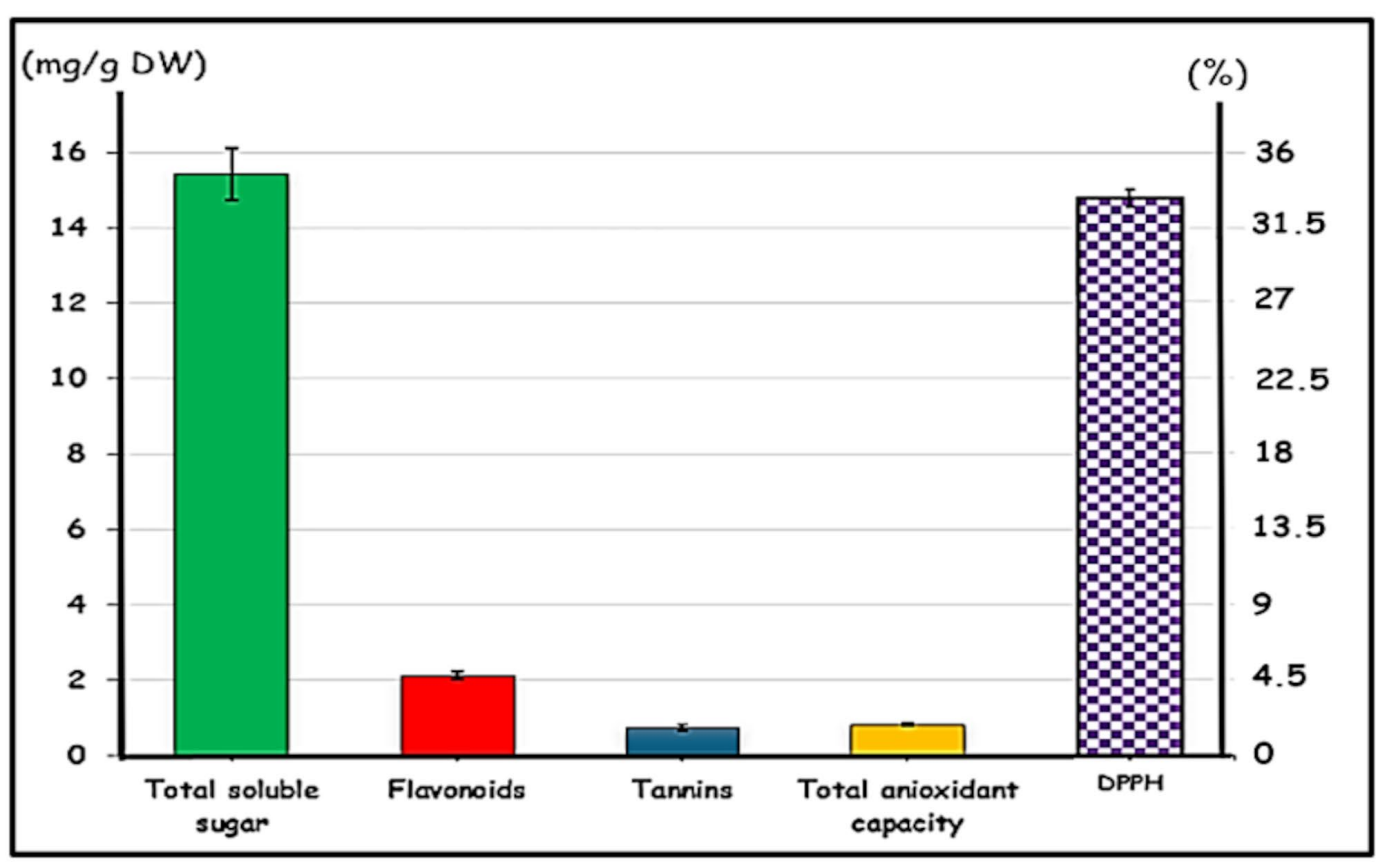
Phytochemicals of 50% *Cupressus sempervirens* ethanolic extract

The ethanolic extract of *C. sempervirens* leaves at 50% concentration revealed the presence of bioactive metabolites: flavonoids, tannins, and carbohydrates (TSS). It was noted that the extract contains 15.43 ± 0.675 mg soluble sugar, 2.13 ± 0.1 mg flavonoids, 0.739 ± 0.087 mg total tannins per g dry matter (Fig. 1), whereas the total antioxidant capacity reached 0.824 ± 0.043 mg/g (Fig. 1).

As for the DPPH radical-scavenging procedure, the 50% ethanolic extract of the plant showed that the potential free-radical activity (DPPH) reached 33.48%±0.591 (Fig. 1).

### Gas chromatography-mass spectrometry (GC-MS) analysis

**Fig. 2:**
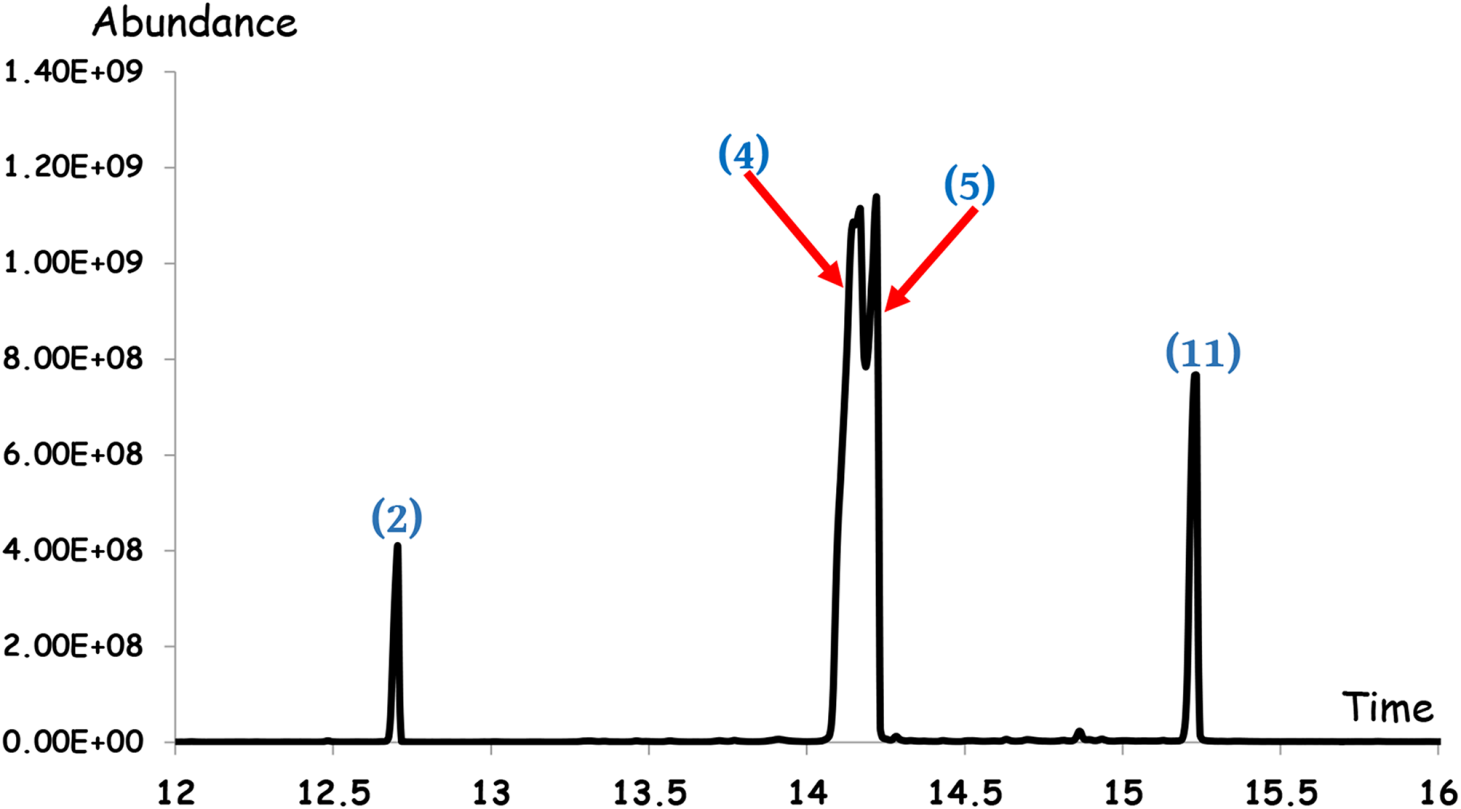
Chemical constitutes of *Cupressus sempervirens*, showing the four major essential oils indicated by numbers above the peaks (according to Table 1): **2.** Palmitic Acid (at 12.7 min.), **4.** Eicosapentaenoic acid (at 14.1 min.), **5.** 10,12-Docosadiynedioic acid (at 14.2 min.), **11.** 10-Undecynoic acid (at 15.2 min.)

Chemical constituents of essential oil (fatty acids and their derivatives) extracted by petroleum ether are listed in (Table 1), and their identification was based on relative time (minutes), percentage of total content, molecular weight (Dalton), and molecular formula. Analysis of *C. sempervirens* showed 13 types of essential oils (FAs). The major compounds found in the extracted oil were four i.e., Eicosapentaenoic acid (50.85%), 10,12-Docosadiynedioic acid (27.58%),10-Undecynoic acid (14.28%) and Palmitic Acid (5.42%); the rest 9 compounds concentrations in the extract ranged from 0.04 to 0.6% (Table 1; Fig. 2).

**Table 1:** Chemical constituents of essential oil obtained from *Cupressus sempervirens*

No.	Compounds	RT (min.)	Peak area (%)	Molecular weight	Molecular formula
1	Myristic acid, TMS derivative	11.7321	0.0404	300.248	C_17_H_36_O_2_Si
2	**Palmitic Acid**,** TMS derivative**	12.7048	**5.4221**	328.28	C_19_H_40_O_2_Si
3	Octanoic acid, TMS derivative	13.9122	0.2253	216.155	C_11_H_24_O_2_Si
4	**Eicosapentaenoic acid**,** TMS derivative**	14.1525	**50.8597**	374.6321	C_23_H_38_O_2_Si
5	**10**,**12-Docosadiynedioic acid**,** 2TMS derivative**	14.2211	**27.5845**	506.9	C_28_H_50_O_4_Si2
6	2-Methyloctanoic acid, TMS derivative	14.2841	0.1883	230.17	C_8_H_18_O_2_Si
7	5,8,11-Eicosatrienoic acid, (Z)-, TMS derivative	14.6331	0.0716	378.295	C_23_H_42_O_2_Si
8	2-Ethylhexanoic acid, TMS derivative	14.7018	0.1144	216.155	C_11_H_24_O_2_Si
9	2-Methylpropanoic acid, TMS derivative	14.8677	0.356	118.081	C_7_H_16_O_2_Si
10	Dodecanedioic acid, 2TMS derivative	14.9364	0.0774	374.231	C_18_H_38_O_4_Si_2_
11	**10-Undecynoic acid**,** TMS derivative**	**15.2339**	**14.2843**	256.4564	C_14_H_28_O_2_Si
12	Decanoic acid, TBDMS derivative	16.6644	0.6435	286.233	C_16_H_34_O_2_Si
13	2-Heptenoic acid, octyl ester	18.65	0.1323	240.209	C_15_H_28_O_2_

RT: Retention Time

### Acaricidal activity

#### Mobility and mortality

**Fig. 3:**
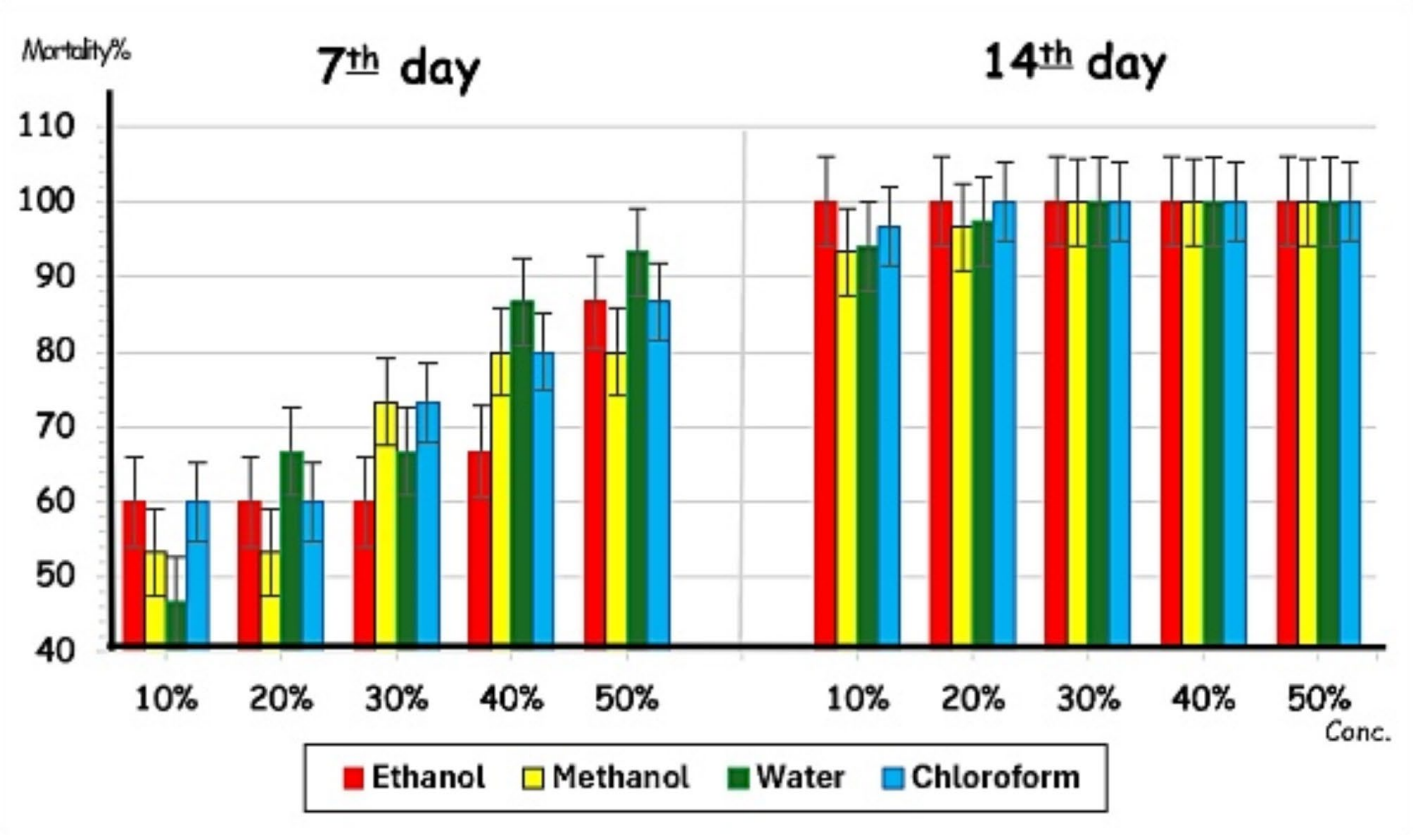
Effect of *Cupressus sempervirens* extracts at 10–50% on the mortality percentage (%) of *Rhipicephalus annulatus* at 7th and 14th after treatment

All semi-engorged females of *R. annulatus* in the control group showed their ability to move (100%) during the experimental period (14 days) and compared with treated ones (Fig. 3). Mobility percentage of females treated with ethanolic extracts of *C. sempervirens* at 10%, 20%, 30%, 40%, and 50% decreased markedly from 1st to 14th day after treatment ranged between 100% and 0% at all concentrations (*p* < 0.001). On the other hand, the percentage of dead ones increased from 0 to 100% using 10%-40%, and 6.7–100% using 50% during the same period versus 0% in the control group (*p* < 0.001) (Fig. 3). By using methanolic extracts, the percentage of mobile ticks decreased from 1st to 14th day after treatment with the same concentrations being 100 − 6.7% (*p* < 0.01), 100 − 3.3% (*p* < 0.001), 100-0% (*p* < 0.001), 100-0% (*p* < 0.001) and 93.3-0% (*p* < 0.001), respectively. Percentage of dead females recorded 0-93.3% (*p* < 0.01), 0-96.7% (*p* < 0.001), 0-100% (*p* < 0.001), 0-100% (*p* < 0.001) and 6.7–100% (*p* < 0.001) using 10-50%, respectively (Fig. 3). Similarly, the percentage of mobile females decreased as the concentration and time increased reaching 6.7% (*p* < 0.01), 3.3% (*p* < 0.001), 0% (*p* < 0.001), 0% (*p* < 0.001) and 0% (*p* < 0.001), respectively after 14 days of treatment with water extracts, while increased in the dead ones recorded 0-93.3% (*p* < 0.01), 0-96.7% (*p* < 0.001), 0-100% (*p* < 0.001), 6.7–100% (*p* < 0.001), and 6.7–100% (*p* < 0.001), respectively in all treated groups within 14 days (Fig. 3). Chloroform extracts showed a decrease in the mobile tick percentage during the same period (100 − 3.3% for 10% (*p* < 0.01), and 100-0% for 20% (*p* < 0.01), 30%, 40% and 50% (*p* < 0.01). Dead female percentages increased from 0 to 96.7% after treatment with 10% (*p* < 0.01) for 14 days, and from 0 to 100% after treatment with 20% (*p* < 0.01), 30%, 40%, and 50% (*p* < 0.001) (Fig. 3). No differences were recorded between treated groups, either mobile or dead females, within each extract type (*p* > 0.05).

In general, the percentage of mobility decreased, and mortality increased directly proportionally with the two factors; days after treatments and concentrations of extracts (both or each factor alone). The ethanolic extracts (10–50%) showed their strongest acaricidal effect, where the mortality percentage reached 100% on the 14th day.

### Lethal concentrations (LC)

The estimated LC_50_ and LC_95_ of *C. sempervirens* ethanol extract were recorded as the lowest values (12.2% and 17%, respectively) after 14 days of treatment. On the other hand, methanol and water extracts recorded the highest LC values being 16.2% LC_50_ and 20.9% LC_95_ for both (Table 2) on the 14th day after treatment.

**Table 2:** Lethal concentrations of *Cupressus sempervirens* at 7th and 14th after treatment *Rhipicephalus annulatus*

Solvents	7 days		14 days	
	LC_50_ (%)	LC_95_ (%)	LC_50_ (%)	LC_95_ (%)
**Ethanol**	30.8	244	12.2	17
**Methanol**	21.2	215.6	16.2	20.9
**Water**	18.2	163.8	16.2	20.9
**Chloroform**	20	198.5	13.6	18.4

### Efficacy

**Fig. 4:**
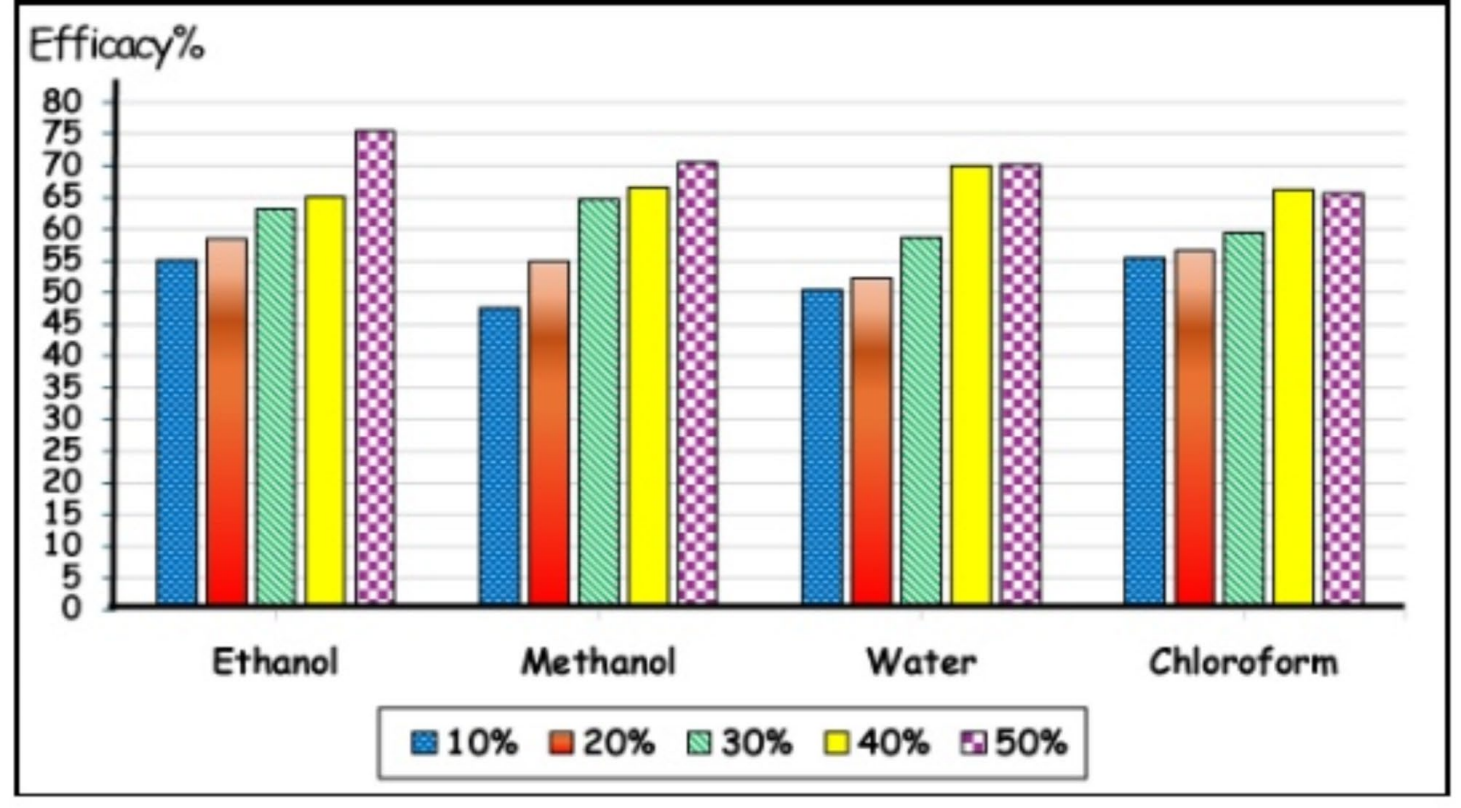
Efficacy of *Cupressus sempervirens* extracts at 10–50% against *Rhipicephalus annulatus* during treatment period

The efficacy of *C. sempervirens* ranged between 55.2 and 75.4%, 47.6–70.5%, 50.5–70.2%, and 55.5–65.7% for ethanol, methanol, water, and chloroform extracts, respectively, after using 10–50% of each (Fig. 4) during the treatment period.

## Discussion

It’s important to point out that, the main objectives of the research in the acaricides field are not only to reduce the concentration of chemicals while enhancing biological activity against organisms, thus reducing the possibility of population resistance, but also to ensure that biochemicals are safe and from a cheaply available source [47]. Despite the problems associated with using chemical acaricides, vaccines, predators, and natural enemies, plants are still the safer, cheaper source, and more effective in controlling ticks.

The present results revealed that the ethanolic extracts (10–50%) showed the strongest acaricidal effect, with mortality reaching 100% after 14 days, the lowest LC_50_ and LC_95_ (12.2% and 17%, respectively), and the highest efficacy (75.4%), compared with other used extract types. So, the current study and most others recommended using plant extracts based on alcohol, especially ethanolic ones. Alcohol’s ability to extract apolar chemical constituents that can more easily penetrate tick cuticles may be the reason for the extract’s higher efficacy [48, 49]. This is consistent with the view expressed by Balashov [50], who suggested that the external tick cuticle wax may cause organic solvents to perform better in acaricidal bioassays. Also, Chagas et al. [51] reported that chemical constituents that are more non-polar can penetrate the cuticle more easily.

Compared to other organic solvents, which are generally less hazardous and safer, ethanol offers several benefits when used as the extraction solvent [52]. Moreover, food and medicine manufacturers utilize ethanol as a solvent since it is safe for humans to consume [53]. In addition, ethanol is a general polar solvent that can extract compounds of interest from vegetal material, such as terpenes and flavonoids [54], proteins, carbohydrates, phenolic derivative antioxidant chemicals [55, 56], glycosides, polyacetylenes, sterols, polyphenols, tannins, and alkaloids [57, 58].

Evidence indicates that the phytochemicals and oils comprise a potential, eco-friendly, and safe source of acaricides [59]. In this connection, numerous investigations worldwide have assessed the acaricidal properties of more than 200 plant species including extracts, essential oils, and phytochemical products by bio-assaying with both larval and adult ticks. Observed mortalities against different ticks’ genera ranged from 5 to 100% according to the plant species, concentrations, and exposure time [59, 60]. So, the plants have good results comparable to those of currently used acaricides [61].

Although there are several pieces of research on secondary chemicals, and essential oils (fatty acids) in all parts of *C. sempervirens* [8, 62, 63] against many microorganisms, the current work is considered the first work that studies the efficiency of *C. sempervirens* against *R. annulatus* in Egypt, with analysis of phytochemical components and essential oils. All these researches showed that *C. sempervirens* components had enhanced the repellency and/or toxicity of microorganisms, insects, and ticks [8, 62, 63] which is consistent with the results of our study. It’s important to notice that the geographic location of the studied plant had a strong significant impact on the chemical composition of its secondary metabolites and essential oils [62].

Per the present results, the 50% ethanolic extract of *C. sempervirens* revealed 15.43 ± 0.675 mg total soluble sugar, 2.13 ± 0.1 mg flavonoids, 0.739 ± 0.087 mg tannins and 0.824 ± 0.043 mg total antioxidant capacity per g of the dry weight. The potential free-radical activity (DPPH) was recorded 33.48 ± 0.591%.

Several in vitro assays revealed that some carbohydrates had insecticidal properties [64]. Kılcı and Altun [65] studied the effect of carbohydrates on nutritional preference and development of *Ephestia kuehniella* Zeller (Lepidoptera: Pyralidae), where proven that increasing the carbohydrate concentration in the diet of *Ephestia kuehniella*, leads to increase the amount of food consumption but the pupa dry weight (which is an indicator of fecundity) is decreased. According to Bernays et al. [66] and Juma et al. [67] carbohydrates are one of the feeding stimulants. Bernays et al. [66] mentioned that insufficient concentration of feeding stimulants is necessary for feeding to stop. Juma et al. [67] reported that sugars are crucial for insect herbivores to accept host plants and continue feeding. Moreover, Hu et al. [64] have employed carbohydrates as insecticides due to these characteristics, they revealed the inhibitory effect on *Bemisia tabaci* (both nymphal and adult survival) significantly reduces with decreasing plant sugar concentrations.

Generally, phenolic compounds such as flavonoids and tannins extracted from the plants have repellent and acaricidal activity against many tick species e.g. *R. annulatus*, *R. decoloratus*, *R. microplus*, and *R. pulchellus* [68–70]. According to El Haddad et al. [71], the flavonoids of *Borago officinalis* extract (i.e. methanol, petroleum ether, diethyl ether, ethyl acetate, and n-butanol) produced an in vitro acaricidal activity against *R. sanguineus* which considerably reduced the oviposition and the hatching rate of the eggs and toxic against newly hatched larvae. Fernandez-Salas et al. [72] assayed four tannin-rich plant extracts namely, *Acacia pennatula*,* Leucaena leucocephala*,* Lysiloma latisiliquum*, and *Piscidia piscipula* that showed acaricidal activity against *R. microplus* larvae (54.8%, 66.79%, 56.0%, and 88.14% respectively), with no effect on adults or egg-laying. On the other hand, tannins had a strong effect on the number of eggs laid and larval hatching rate of the same tick species [73].

According to Al-Rajhi et al. [74], *C. sempervirens* extract exhibited antioxidant activity due to the presence of 18 phenolic and flavonoid components. Notably, there is a direct correlation between phenolic content and antioxidant activities, as well as an inverse correlation with DPPH [74, 75]. The latter relationship is attributed to redox qualities, which are essential for quenching singlet and triplet oxygen and adsorbing and neutralizing free radicals [76]. Similarly, the present study indicated that the 50% ethanolic extract can also work as a primary antioxidant which can be involved in the antioxidant biological activity and effective acaricides [76].

Generally, plant essential oils (FAs) have neurotoxic effects on arthropods [77, 78]. Numerous studies have demonstrated that they can bind to octopamine receptors, inhibit arthropods’ acetylcholinesterase (AChE), and act on gamma-aminobutyric acid (GABA), all of which can have fatal consequences [79–82].

There are many studies on the leaf oil content of *C. sempervirens* and its strong effect on arthropods e.g. *Aedes albopictus*, *Culex quinquefasciatus*, and *Hyalomma scupense* where α-pinene and δ-3-Carene are the major volatile oils in *C. sempervirens* which act as repellency (against harmful insects, ticks), acaricidal and larvicidal activities [8, 62]. Under the current results, the main components were Eicosapentaenoic acid (50.85%), 10,12-Docosadiynedioic acid (27.58%),10-Undecynoic acid (14.28%), and Palmitic acid (5.42%).

Eicosapentaenoic acid (EPA) is one of ω-3 polyunsaturated fatty acids and its occurrence in the studied oil analysis was the highest at 50.8%. The findings according to Madden et al. [83] showed that EPA in the host diet had a significant impact on the tick salivary glands’ ability to assimilate arachidonate, which may change the prostaglandin content of the tick saliva. Prostaglandins of the 2-series are found in tick saliva and are thought to aid in the acquisition of bloodmeal. Furthermore, it increases vasodilation and decreases inflammation by regulating cytokine production, inhibits dendritic cell differentiation, maturation, cytokine production, and T lymphocyte proliferation, and regulates the migratory activities of fibroblast and macrophage [84]. So, the presence of EPA with a high concentration in the studied plant could affect Prostaglandins in the silva of *R. annulatus* and lead to altering many of its vital activities.

10,12-Docosadiynedioic acid is one of the fatty acid compounds [85]. It is present in numerous plants e.g. *Ardisia solanacea*, *Lepidium meyenii*, *Manihot esculenta*, *Ricinus communis*,* Withania somnifera* [85–89]. Some studies have examined the biological activities of 10,12-Docosadiynedioic acid as one of the active plant components against microorganisms e.g. *Pseudoperonospora cubensis* (fungus), resistant *Salmonella typhi* (bacteria), besides its activities as one of antioxidant, anti-inflammatory and insect antifeeding compounds which are found in *Ardisia solanacea* [88].

10-Undecylenic acid is a saturated medium-chain fatty acid. It is an undecenoic acid having its double bond in the 10-position and one of (*R*)-Ricinoleic Acid derivatives [90]. 10-Undecenoic acid and its salts are industrially important compounds that are known for their wide biological activities e.g. antifungal, antibacterial, mosquito larvicide, and possess therapeutic potential [90–93]. Furthermore, many studies were conducted examining the high efficiency of undecylenic acid as one of various compounds applied against *R. sanguineus* and *Amblyomma variegatum* ticks [90, 94].

Palmitic acid is one of the most famous saturated fatty acids, which is present in most plants and has various documented biological activities [95–97]. Moreover its acaricidal efficiency against different types of ticks (larvae and adults) e.g. *R. microplus* and *Hyalomma scupense* documented as one of the oils found in many plants e.g. *Carthamus tinctorius*, *Citrus limetta*, *Gossypium* Sp., *Mauritiella armata*, *Mauritia flexuosa*, and *Nasturtium officinale* [8, 82, 98–100].

Although this paper provides a safe, effective, cheap, and available acaricide, it lacks further analysis of the phytochemical compounds with different polarities to compare their biological activities and test the oils for their acaricidal activity against *R. annulatus*. Therefore, in future work, we plan to conduct many studies and analyses on this plant as acaricide.

## Conclusions and future work

Generally, the efficacy of *C. sempervirens* extracts reached 75.4% against *R. annulatus* which is a significant percentage in bio-acaricides. The present work revealed that the ethanolic extracts (at all concentrations 10–50%) showed the strongest acaricidal effect compared with other used extract types; notably at 50% had the highest acaricide effect compared with other ethanolic extract concentrations. The information provided in this study leads one to conclude that secondary metabolites and essential oils (fatty acids) found in *C. sempervirens* products may provide a different approach to managing tick populations that are either vulnerable or resistant to commercial acaricides. Finally, we strongly recommend using ethanolic extract of *C. sempervirens* against the studied ticks, also we recommend further tests and analysis on the extracted oils and phytochemicals (in different polarities solvents) presented in the studied plant and their effects in biological control against ticks of different species.

## Data Availability

No datasets were generated or analysed during the current study.
